# Genome Sequence of Clinical Acinetobacter baumannii Strain V15

**DOI:** 10.1128/mra.00169-23

**Published:** 2023-06-26

**Authors:** Valentín Permingeat, Bárbara Perez Mora, Mariano Torres Manno, Rocío Giordano, Natalia Arana, Gabriela Müller, Renatas Krasauskas, María Alejandra Mussi

**Affiliations:** a Centro de Estudios Fotosintéticos y Bioquímicos (CEFOBI-CONICET), Universidad Nacional de Rosario (UNR), Rosario, Argentina; b Institute of Biosciences, Life Sciences Center, Vilnius University, Vilnius, Lithuania; c Institute of Biotechnology, Life Sciences Center, Vilnius University, Vilnius, Lithuania; University of Maryland School of Medicine

## Abstract

Acinetobacter baumannii is recognized as a critical human pathogen by the World Health Organization, and therefore there is increasing interest in studying its biology and pathophysiology. Among other strains, A. baumannii V15 has been extensively used for these purposes. Here, the genome sequence of A. baumannii V15 is presented.

## ANNOUNCEMENT

In the last 50 years, the world has experienced the emergence and rapid spread of a new plague: microbes resistant to multiple antibiotics, preventing the treatment of many diseases (1–3). Of these microbes, A. baumannii has been recognized by the World Health Organization (WHO) as a critical pathogen (4).

A. baumannii strain V15 presents differential characteristics with respect to domesticated strains ATCC 17978 and ATCC 19606. V15 forms robust biofilms, produces a capsule, and generates large amounts of extracellular compounds (5). Moreover, it responds to light at both environmental temperatures and those of warm-blooded hosts (6). Furthermore, V15 is sensitive to multiple antibiotics and genetically manipulable.

Here, we present the draft genome sequence of A. baumannii V15, which was isolated in 2010 from a patient in the Toxicology Unit of the Vilnius University Emergency Hospital, Lithuania (7). Initial identification was performed using the Phoenix system (BD, USA) and amplified rDNA gene restriction analysis (7). For sequencing, a single colony of V15 was inoculated into 3 mL LB broth medium and grown at 37°C for 18 h. The cells were then pelleted and used for DNA extraction using a commercial kit (Macherey-Nagel, Allentown, USA) (8).

The genome of V15 was sequenced using the Illumina NovaSeq 6000 platform at Novogene Co. Briefly, genomic DNA was randomly sheared into short fragments, which were then end-repaired, A-tailed, and further ligated with Illumina adapters. The fragments with adapters were PCR amplified, size selected, and purified. The library was checked using Qubit and real-time PCR for quantification and Bioanalyzer for size distribution detection. The quantified libraries were pooled and sequenced on Illumina platforms, with quality parameters (Q scores) between Q20 and Q30. The sequence quality distribution was ascertained by a Phred score higher than 35, and the error distribution was normal for Illumina sequences. Read quality control, adapter trimming, and sequence filtering were performed using fastp v0.23.3 software with default parameters (8).

Sequencing yielded 7,884,616 paired-end reads of 150 bp, which were assembled into 44 contigs using the Read Assembly and Annotation Pipeline Tool (RAPT) v0.5.4 (rapt-41589182; SKESA v2.5.0; PGAPX v2022-10-03.build6384; https://www.ncbi.nlm.nih.gov/rapt/). Contigs 1 and 2 corresponded to two complete plasmids named pV15_1 and pV15_2, respectively. The resulting assembly yielded *N*_50_ and *L*_50_ values of 194,502 bp and 8, respectively.

The complete genome size of A. baumannii V15 is 3,863,035 bp, including the chromosome and plasmids. V15 has one chromosome of 3,786,160 bp, encoding 3,610 predicted coding sequences (CDSs), with a GC content of 38.5%. Plasmid pV15_1 contains 68,113 bp, with a GC content of 33%, while pV15_2 has 8,762 bp, with a GC content of 38%. The plasmids encode 87 and 12 predicted CDSs, respectively.

Average nucleotide identity (ANI) values were obtained between V15 and type strains ATCC 17978, ATCC 19606, and UPAB1 using the ANI Calculator platform (OrthoANIu v1.2) (9). The ANI values were found to be 97.92%, 97.98%, and 97.80%, respectively.

The sequences and contigs were assembled using SKESA (10) (within the RAPT pipeline). Open reading frames (ORFs) were predicted using the Prokaryotic Genome Annotation Pipeline (PGAP) (11) (within the RAPT pipeline). Default parameters were used for all software unless otherwise specified.

### Data availability.

This whole-genome shotgun project has been deposited at DDBJ/ENA/GenBank under accession number JAQSVA000000000. The version described in this paper is version JAQSVA010000000. The raw reads were submitted to the NCBI SRA under accession number SRR23455319, BioProject accession number PRJNA931904, and BioSample accession number SAMN33101523. The GenBank accession numbers for pV15_1 and pV15_2 are JAQSVA010000035 and JAQSVA010000036, respectively. The GenBank accession numbers of the type strain genome sequences used for comparison are GCF_029459615.1, GCF_009035845.1, and GCF_006843645.1 for ATCC 17978, ATCC 19606, and UPAB1, respectively.
